# Temperature and Thermal Diffusivity Diagnostics in Laminar Methane Flames Using Infrared Four-Wave Mixing Techniques

**DOI:** 10.1177/00037028241233609

**Published:** 2024-02-26

**Authors:** Zihao Song, Xing Chao, Anna-Lena Sahlberg

**Affiliations:** 1 538782 School of Aerospace Engineering, Tsinghua University, Beijing, China; 2 Combustion Physics, Department of Physics, 5193 Lund University, Lund, Sweden; 3 539666 Center for Combustion Energy, Tsinghua University, Beijing, China

**Keywords:** Flames thermometry, infrared degenerate four-wave mixing, IR-DFWM, infrared laser-induced grating spectroscopy, IR LIGS, thermal diffusivity measurement.

## Abstract

Four-wave mixing techniques, such as coherent anti-Stokes Raman spectroscopy (CARS), laser-induced grating spectroscopy (LIGS), and degenerate four-wave mixing (DFWM), have been widely used in combustion diagnostics due to their advantages of high signal-to-noise ratio (S/N), coherent signal, and spatial resolution. In this work, a nano-second pulsed laser is utilized to generate mid-infrared (near 3 µm) pump beams, exciting the rovibrational transitions of nascent water in flames. Combined LIGS and DFWM measurements are demonstrated in premixed laminar CH_4_/O_2_/N_2_ flames with equivalence ratios from 0.6 to 1.5, to achieve precise thermometry in a wide range of flame conditions. The flame temperatures were also measured by thermocouple as a reference, and the results from LIGS and DFWM align well with the trends shown in the thermocouple measurements. In fuel-lean flames, where the mass-to-specific-heat ratio variation is minimal, LIGS provides temperature data with a precision better than 16 K (0.8%). In fuel-rich flames, where the increased H_2_ concentration in the flame introduces uncertainty in gas constants thus affecting the accuracy of LIGS thermometry, DFWM is instead employed for temperature measurement since it is less sensitive to the gas composition within the measured volume. The high-precision LIGS temperatures in lean flames serve as temperature reference during the DFWM calibration of the degree of saturation, and a precision better than 90 K (4.5%) is achieved for DFWM thermometry. In addition to temperature, a theoretical model is employed to fit LIGS signal time waveforms, extracting the local speed of sound and thermal diffusivity with precisions better than 0.5% and 1.3%, respectively. These high-precision measurements contribute additional data for flame research and simulation calculations. This way, the combined use of DFWM and LIGS proves the potential for accurate thermometry and diagnostics of other thermodynamic parameters across a wide range of flame conditions.

## Introduction

Laser techniques, with the advantages of non-intrusive diagnostics, high spatial and temporal resolution, and the potential for simultaneous measurement of multiple parameters, have been widely used to improve the understanding of combustion physics and reaction mechanisms.^1^ Temperature, as one of the most important flame characteristics, has led to the development of many laser-based techniques for accurate flame thermometry, such as laser absorption spectroscopy,^2^ laser-induced fluorescence,^3^ and Rayleigh scattering.^4^ In addition to these linear methods, the nonlinear four-wave mixing techniques, such as coherent anti-Stokes Raman spectroscopy (CARS),^5^ degenerate four-wave mixing (DFWM),^6^ and laser-induced grating spectroscopy (LIGS),^7^ have been demonstrated for flame thermometry in a wide range of applications with the advantages of the coherent nature of the signal and reduced sensitivity to collisional quenching.

Most nonlinear techniques have previously been applied using pulsed lasers in the ultraviolet visible (UV–Vis) spectral range.^8–12^ However, in recent years, with the development of infrared (IR) lasers and detectors, infrared nonlinear techniques have been developed to detect many important fuel molecules and intermediate species in combustion that lack accessible transitions in the UV–Vis range, such as methane (CH_4_),^13,14^ acetylene (C_2_H_2_),^15^ hydrochloric acid (HCl),^15^ ethylene (C_2_H_4_),^16,17^ propane (C_3_H_8_),^18–20^ sulfur hexafluoride (SF_6_),^16^ ammonia (NH_3_),^17^ water (H_2_O),^21–23^ and carbon dioxide (CO_2_),^19,24,25^ using two-photon Raman excitation, direct excitation of fundamental vibrational bands using mid-infrared lasers, or excitation of high vibrational overtones in near-infrared regions. With reference to these resonant excitation schemes, laser-induced thermal grating spectroscopy (LITGS) thermometry can be designed for different applications with different species inside. Although the non-resonant version of LIGS, namely laser-induced electrostrictive grating spectroscopy (LIEGS), is also widely used for temperature measurements because it does not require absorbing species during the measurement or excitation laser with a specific wavelength,^26,27^ LITGS thermometry is the topic of this work and mainly discussed here. Flame thermometry using infrared laser-induced thermal grating spectroscopy (IR-LITGS) of H_2_O near 3 μm was demonstrated in C_2_H_4_/air flames^28^ and CH_4_/H_2_/air flames,^29^ and a repetitive single-shot temperature precision better than 1% was achieved thanks to the high signal-to-noise ratio (S/N) LITGS signals arise from the abundant distribution of H_2_O in hydro-carbon flames and strong absorption line of H_2_O near 3 μm. However, as noted in the literature,^28^ thermometry using IR-LITGS in fuel-rich hydrocarbon flames needs to be carefully considered since the increasing H_2_ concentration in the product zone of rich flames has a great influence on the specific heat-to-mass ratio γ/*M*. This increases the uncertainty in the gas constants and thus limits the accuracy of inferring the flame temperature from the measured speed of sound *c*_s_. Sun et al.^30^ performed IR-DFWM measurements targeting H_2_O molecules in CH_4_ flames and temperatures were derived from the DFWM signal intensity ratio between the two line-groups around 3231 cm^–1^. High S/N DFWM spectrum was obtained thanks to the “laser-like” signal beam which ensures the detector can be placed away from the flames to reduce the thermal infrared radiation and the target species H_2_O present in relatively high concentrations in CH_4_ flames. Due to the high sensitivity of the chosen water lines, the DFWM thermometry can potentially achieve very high precision. However, since DFWM thermometry is highly sensitive to the degree of saturation in the flames,^31^ this method requires a calibration of the DFWM signal intensity ratios versus quantitative temperatures in known conditions to determine the function between the signal intensity ratio and temperature.

Comparing the two thermometry techniques, LITGS is a high-precision calibration-free technique suitable for diagnostics where the flame composition is known, particularly in lean flames. DFWM is more suitable for rich flame thermometry or thermometry in unknown flame conditions since this method does not depend on the flame composition within the measured volume. In previous works,^30,31^ laser Rayleigh scattering was used to provide temperatures during DFWM calibration with an uncertainty of 7%. To further improve the performance of DFWM thermometry, a method that provides more accurate and precise temperatures is needed during the calibration process. Using flames with known flame composition, LITGS can be used for calibration of the DFWM intensity ratio for the relevant temperature intervals. This improves the precision of the calibration, and in an additional benefit, LITGS and DFWM can be implemented simultaneously using the same pump–laser system.^32^

In this work, combined IR-DFWM/LITGS measurements targeting H_2_O (near 3231 cm^–1^) are performed in atmospheric laminar CH_4_/air flames (equivalence ratios from 0.6 to 1.5). The single point temperatures in fuel-lean methane flame conditions were measured by IR-LITGS with a precision better than 0.8% (16 K). Taking such precise temperatures in lean flames as prior information during DFWM calibration, the flame temperatures in fuel-rich flames were then measured using IR-DFWM with a precision of 4.5% (90 K), demonstrating the combined IR-DFWM/LITGS thermometry as a promising temperature measurement method in both fuel lean and rich flames. In addition to flame temperatures, other thermodynamic parameters such as speed of sound and thermal diffusivity in each flame were inferred from the temporal LITGS signal waveform with precisions better than 0.5% and 1.3%, respectively, providing further data for flame research and simulation calculations. The temporal resolution of this combined IR-DFWM/LITGS, limited by the wavelength scanning time of minutes required in DFWM, means it is not suitable for applications in turbulent flames. However, in addition to applications in laminar flames, the combined IR-DFWM/LITGS can be potentially used in quasi-stable combustion situations, where the combustion environment is slowly varying over time, such as biomass combustion where the temperature varies over several minutes from ignition to when the fuel has been consumed.^6^ The ability of DFWM quantitative concentration measurements also brings the possibility of IR-DFWM/LITGS thermometry being implemented together with concentration measurements of minor species, radicals, and pollutants in laminar flames,^6^ which is valuable for chemical modeling and the development of new biofuels.^33^

## Theory

### Theory of LIGS and Simulation of Signal Waveform

The fundamental theory of LIGS can be found in Kiefer and Ewart^8^ and Hemmerling et al.,^34^ and only a brief description is included here. Two pump laser beams with the same wavelength of λ_pump_ crossing at an angle θ create an interference pattern, an intensity fringe pattern with characteristic grating spacing Λ = λ_pump_/(2sin(θ/2)). When the pump beams are resonant with molecular transitions present in the crossing volume, the spatially periodic intensity pattern will lead to a spatial modulation of the excited molecular state population. Collisional quenching redistributes the laser energy absorbed by the excited molecules into heat, leading to a spatially modulated refractive index distribution with the same grating spacing Λ, i.e., a laser-induced thermal grating (LITG) is formed. The temporal behavior of this thermal grating reflects the superposition of a stationary temperature grating (decays exponentially due to thermal diffusion) and a standing wave oscillation (caused by the density modulation due to the rapid change of temperature and gas density in the LITG, which decays as the acoustic waves travel out of the measurement volume). The standing wave oscillates at the modulation frequency *f*_osc_ = *c*_s_/Λ, where *c*_s_ is the speed of sound within the measured volume. In addition to the resonant grating, this density modulation, thus standing wave oscillation can also be generated by non-resonant pump laser beams due to the presence of a strong electric field gradient modulating the gas density through electrostriction. However, this process requires higher pump laser energies (typically 10–100 times stronger compared to resonant pump laser beams) and is usually negligible in cases where there is strong resonant absorption.^28,35^ In this work, resonant pump laser beams are utilized and the enhanced LITG is generated.

The formation and evaluation of the LITG result from the processes of laser excitation, excitation and thermal energy deposition, density perturbation, and its associated modulation of the refractive index. Thermodynamic parameters such as the speed of sound, thermal diffusivity, and thermal energy transfer rate, will characterize the grating evaluation and thus affect the temporal signal waveform. The dynamic evaluation of the LITG can be probed by the laser-like signal beam, which is the scatter of the probe beam aligned to cross the induced LITG at the Bragg angle. Abundant information can be revealed from the signal intensity level, oscillation frequency, and signal temporal waveform. It is also possible to generate scattering signals by aligning the probe laser at higher-order Bragg angles. If the LITG forms a perfect sinusoidal grating, there would be no scattering at higher-order Bragg angles; however, if the absorption of the pump beams is enough to saturate the LITG, it can generate overtones in the shape of the LITG, making scattering at higher-order Bragg angles possible, although the efficiency of the scattering will be reduced compared to the first-order Bragg angle. However, it is a viable option in this paper, where the first-order Bragg angle is so small that the practical limitations of the setup make it very difficult to separate the probe and signal beams for interference-free signal detection.

To derive these thermodynamic parameters from the experimental LIGS signal quantitatively, the theoretical description of the LIGS signal waveform is needed. Analytical descriptions for the time evolution of a laser-induced grating can be found in, e.g., the works of Cummings,^36^ Cummings et al.,^37^ Paul et al.,^38^ and Hemmerling and Kozlov.^39^ In this work, Hemmerling's theory is utilized to fit the measured LITGS signals, with the modification of neglecting the signal contribution from electrostriction and the viscous damping. This is because the infrared laser pulse energy is relatively weak, and the viscous damping is slow enough to be negligible compared to the acoustic transit. Considering the above modification, the signal intensity *S*_LIGS_ is given by

(1) $$\begin{array}{c} S_{\mathrm{LIGS}}(t)\propto {\left[S_{i}(t)+S_{f}(t)\right]}^{2} \end{array}$$

(2) $$\begin{array}{c} S_{i}(t)\;=\;M_{i}\left[\cos\;(2\pi f_{\mathrm{osc}}t)\exp\;\left(-\frac{t^{2}}{\tau_{\mathrm{tr}}^{2}}\right)-\exp\;\left(-\frac{t}{\tau_{\mathrm{th}}}\right)\right]\; \end{array}$$

(3) $$\begin{array}{r} \begin{array}{cc} S_{f}(t)\;=\;\\ & M_{f}\left\{\frac{\sin\;(2\pi f_{\mathrm{osc}}t)\exp\;(-t^{2}/\tau_{\mathrm{tr}}^{2})}{2\pi f_{\mathrm{osc}}t\tau_{f}}-\frac{\left[\exp\;(-t/\tau_{\mathrm{th}})-\exp\;(-t/\tau_{f})\right]}{1-(\tau_{f}/\tau_{\mathrm{th}})}\right\} \end{array} \end{array}$$

where the constants *M*_i_ and *M*_f_ represent the relative contributions of “instantaneous” and “fast” energy transfer processes, respectively. The acoustic transit time τ_tr_ describes the lifetime of the acoustic standing wave as the counter-propagating sound waves travel out of the crossing volume, which depends on the beam radius *w* at the measured volume and the speed of sound *c*_s_ as τ_tr _= *w*/( $\sqrt{2}$ *c*_s_). The time constant for the thermal diffusion decay of the LITG, τ_th_, depends on the thermal diffusivity α = κ/(ρ*c*_p_) as τ_th _= (Λ/2π)^2^α^–1^, where κ is the thermal conductivity, ρ is the gas density, and *c*_p_ is the specific heat capacity at constant pressure. τ_f_ is the characteristic time constant of the fast energy transfer process. By fitting the measured LITGS signal temporal waveform with this theoretical model, the oscillation frequency *f*_osc_ and time constants such as τ_tr_, τ_th_, and τ_f_ can be obtained and analyzed.

### Thermometry Using Degenerate Four-Wave Mixing

A detailed theory of DFWM can be found in Abrams and Lind,^40^ and only the conclusions from their model used in this work are briefly introduced here. Abrams and Lind derived an analytical solution for DFWM signal intensity *I*_sig_ by assuming a phase-conjugate geometry alignment, as well as stationary two-level absorbers, an optically thin medium, and monochromatic laser beams, all the beams being polarized in the same direction and the probe beam having non-saturating intensity, which can be expressed as:

(4) $$\begin{array}{c} I_{\mathrm{sig}}\;=\;\frac{\alpha_{0}^{2}L^{2}}{1+\delta^{2}}\frac{4{\left(I_{\mathrm{pump}}/I_{\mathrm{sat}}\right)}^{2}}{{\left(1+4I_{\mathrm{pump}}/I_{\mathrm{sat}}\right)}^{3}}I_{\mathrm{probe}} \end{array}$$

where the *I*_pump_ and *I*_probe_ are the intensity of the pump laser and probe laser, respectively. In this work, *I*_pump_ ≈ *I*_probe_ since the pump and probe lasers are close to equally split by BoxCARS plates. δ = *T*_2_(ω – ω_0_) is the normalized detuning factor, where *T*_2_ is the rate of coherence dephasing. *I*_sat _= (1+δ^2^) ℏ cɛ_0_/(2*T*_1_*T*_2_μ^2^) is the saturation intensity, where *T*_1_ is the population dephasing rate, µ is the transition dipole moment, ℏ is the reduced Planck constant, and *c* and ɛ_0_ are the speed of light and permittivity in vacuum, respectively. *L* is the length of the interaction region and α_0_ = (μ^2^Δ*N*_0_*kT*_2_)/(2ɛ_0_ ℏ ) is the line center absorption coefficient, where Δ*N*_0_ is the population difference between the upper and lower states in the absence of an applied field and *k* is the magnitude of the wave vector.

Although Eq. 4 is based on many assumptions that may not always be satisfied under practical experimental conditions, it is fairly successful in analyzing the experimental DFWM signal intensity in many situations.^6,15^ Therefore, in this work, a simplified empirical equation is used to calculate the DFWM signal intensity, based on Abrams and Lind's model and previous measurements of DFWM signal intensity:^30,41^

(5) $$\begin{array}{c} I_{\mathrm{sig}}(\nu )\propto \sigma {\left(\nu \right)}^{2}N^{2}\propto {\left[\underset{ij}{\sum }\;S_{ij}(T)g(\nu )\right]}^{2}N^{2}\; \end{array}$$

where *S_ij_*(*T*) is the absorption line strength of transition *ij* at temperature *T* and *g*(ν) is the normalized absorption line shape function.

The temperature dependence of the line strength *S*_ij_(*T*) can be expressed as:

(6) $$\begin{array}{c} S_{ij}(T)\;=\;S_{ij}(T_{\mathrm{ref}})\frac{Q(T_{\mathrm{ref}})}{Q(T)}\frac{\exp\;(-c_{2}E^{\prime \prime }/T)}{\exp\;(-c_{2}E^{\prime \prime }/T_{\mathrm{ref}})}\frac{1-\exp\;(-c_{2}v_{ij}/T)}{1-\exp\;(-c_{2}v_{ij}/T_{\mathrm{ref}})} \end{array}$$

where *T*_ref_ = 296 K is the reference temperature, *Q*(*T*) is the total internal partition sums at temperature *T*, *c*_2_ is the second radiation constant, and *E*′′ and ν*_ij_* are the lower state energy and frequency of the transition, respectively. As different absorption lines can have different lower state energy *E'*′, different lines show a different temperature dependence. Therefore, the temperature can be deduced from the relative intensity of different absorption lines in one measured DFWM spectrum *I*_sig_(ν) of the selected species. Considering the sensitivity of temperature measurement, which is enhanced when the two transitions used for intensity ratio have larger differences in their lower-state energy, two high-temperature H_2_O absorption line groups near 3231 cm^–1^ (lines information shown in Table I) are chosen for DFWM thermometry. Additionally, the H_2_O lines in this spectral region are free from interference from CO_2_ and CO. The line strength of each H_2_O line can be measured either by scanning the laser over the two absorption lines or placing the laser on the peak of each absorption line for a short time. The scanning method has been chosen over the on-line approach since the former leads to more reliable measurement results^31^ and the scanning method is suitable for the stable laminar flames that were studied here.

**Table I.** Hot water lines information near 3231 cm^–1^ from HITEMP 2010.^42^

Line Group	Transition wavenumber, ν (cm^–1^)	Line intensity at 1800 K, *S*_ij_ (cm·molecule^–1^)	Lower-state energy of transition, *E*'’ (cm^–1^)
I	3230.9830	6.79 × 10^–22^	1789.04
I	3230.9833	20.17 × 10^–22^	1789.04
II	3231.3206	19.43 × 10^–22^	5035.13
II	3231.3316	7.31 × 10^–22^	5713.25
II	3231.3316	21.95 × 10^–22^	5713.25

Equation 4 shows that the DFWM signal intensity depends on the degree of excitation saturation. In the saturation region where *I*_pump _≥ *I*_sat_, the signal intensity *I*_sig_ ∝ μ^2^*N*^2^*L*^2^*T*_2_/*T*_1_ is less sensitive to laser intensity fluctuation and collisional quenching effects, and is generally preferred in practical measurements. However, it is critical to note that the saturation intensity *I*_sat_ is varying for different transitions when performing DFWM spectrum scanning because of the different transition dipole moments. The degrees of saturation for the two H_2_O line groups I and II are often different and affected by the experimental arrangement and laser energy level, and the signal intensity ratio versus temperature thus becomes dependent on the exact degree of saturation of each line. Since the signal intensity ratio for the two H_2_O lines at different degrees of saturation is difficult to simulate with simple models, a calibration of the function between signal intensity ratio and temperature is required for accurate DFWM thermometry. To achieve that, the accurate temperature measurements in lean flames using LITGS are used as reference temperature for the DFWM line ratios, and the detailed discussion of this process can be found in the following contents. The advantage of DFWM thermometry over LITGS is that prior information on composition species is not needed, thus it is more applicable in situations where the gas composition is difficult to predict, such as in fuel-rich flames.

## Experimental

### Materials and Methods

**Figure 1.**
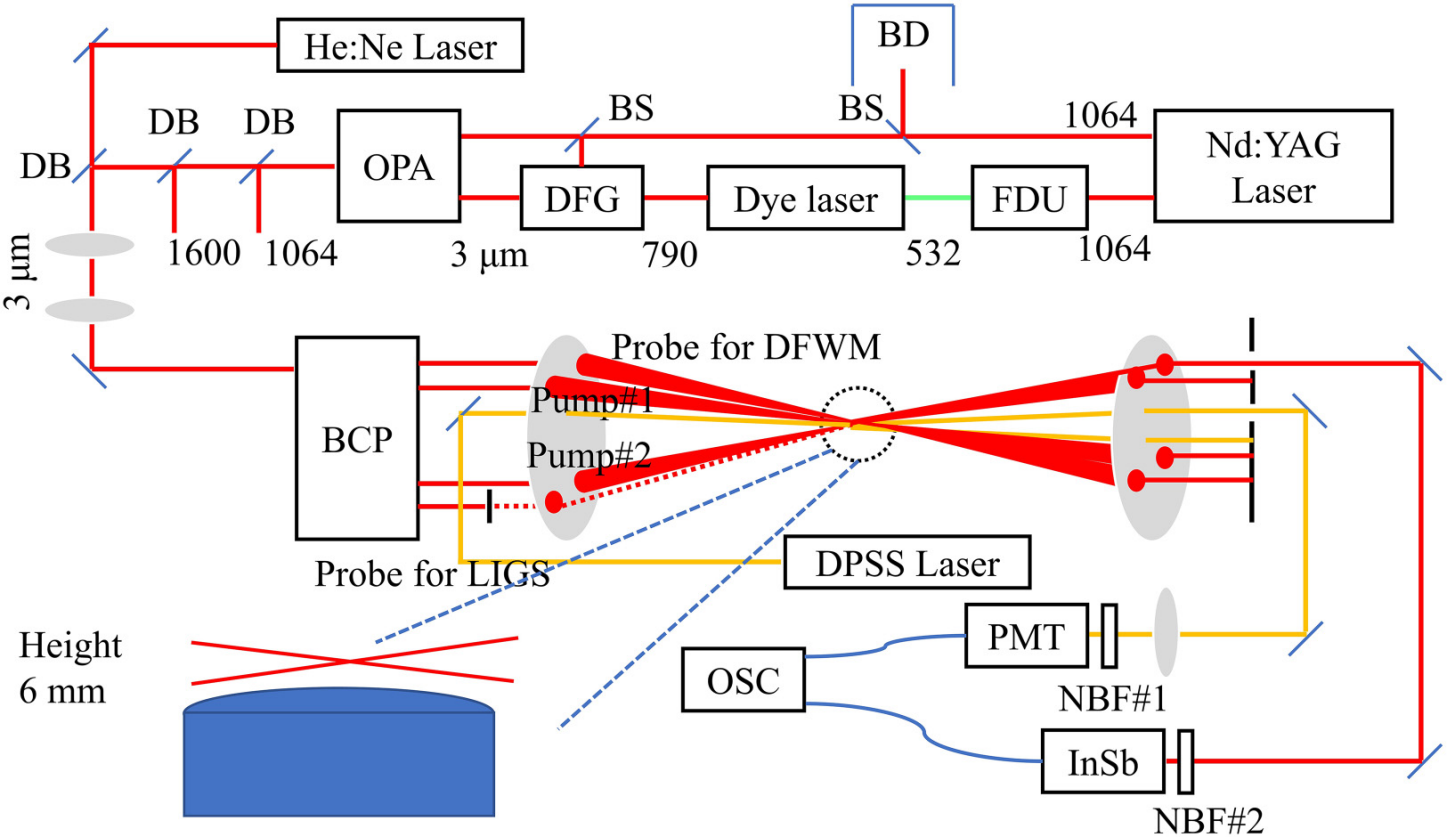
Schematic of IR-DFWM/LITGS experimental setup. FDU: frequency doubling unit, BD: beam dump, BS: beam splitter, DFG: difference frequency generator, OPA: optical parameter amplifier, DB: dichromatic beam splitters, BCP: BoxCARS plates, DPSS: diode pump solid state, PMT: photomultiplier tube, OSC: oscilloscope, NBF: narrow bandpass filter.

The IR-DFWM/LITGS experimental setup used in this work is shown in Figure 1. The LITGS signal was first recorded with the laser wavelength set at the peak of line group I at 3230.98 cm^–1^ and after this, the DFWM excitation scan was recorded in the same flame. Since stable laminar flames are studied in this work, the LITGS and DFWM were not performed simultaneously here for simplicity. In situations where the combustion is slowly varying over time, such as biomass combustion where the temperature varies over several minutes from ignition to when the fuel has been consumed,^6^ it is possible to record the single-shot LITGS signals simultaneously with either the DFWM excitation scan or the on-line DFWM thermometry approach.^32^ However, this simultaneous DFWM/LITGS temperature measurement can hardly be used for turbulent flames, since the temporal resolution is mainly limited by the wavelength scanning time required in DFWM.

### Laser Diagnostics System

A tunable pulsed laser near 3 µm is used for IR-DFWM and as the pump laser for IR-LITGS, producing laser pulses with a repetition rate of 10 Hz and single pulse energy of 6–8 mJ, and full width half-maximum (FWHM) linewidth estimated to be 0.025 cm^–1^.^24^ A more detailed description of this infrared laser system can be found in the previous literature.^28^ For IR-DFWM, four parallel laser beams with equal intensity were generated using two BCARS plates.^15^ The two pump lasers and one probe laser were focused on one single point using a CaF_2_ lens with a focal length of +500 mm, giving a probe volume of 0.2(width) × 0.2(height) × 10(length) mm^3^. The IR-DFWM signal propagates in a direction satisfying phase-matching and is collected by an InSb detector (Teledyne Judson Technologies, J10D-M204-R04M-60) with a PA-9 transimpedance pre-amplifier. In this work, no infrared bandpass filter was used since the interference from flame radiation is minor after the 4 m length path to the detector. For IR-LITGS, pump nos. 1 and 2 were used as the infrared pump beams for LITGS signal generation, and a continuous-wave laser (Laserglow Tech., LRS-0671) with a wavelength of 671 nm and an output power of 1000 mW was used as probe laser, which was aligned to cross the grating at the second-order Bragg angle. It is a choice by necessity since the optical layout of probe beam for first-order Bragg angle is limited by the non-adjustable short distance between the two pump beams, which is determined by the thickness of BoxCARS plates used to split the pump beams. Although the LITGS signal using second-order scatter is expected to be much weaker than using first-order scatter (approximately two orders of magnitude), in this work, sufficiently high S/N LITGS signals are obtained in all flames using the second-order Bragg angle. There is no requirement for the wavelength of the probe laser, however, the probe laser duration should be long enough to cover the formation and temporal evolution of the grating. Also, the signal intensity increases linearly with the probe intensity, thus higher probe laser power is needed to improve the S/N. Continuous-wave lasers are easier to obtain and have better power stability than long-pulse lasers, thus it is used in this work. The visible signal scattered from the LITG is collected by a photomultiplier tube (PMT, Hamamatsu H6780-04) with an optical filter (FL670-10, Thorlabs Inc., center at 670 ± 2 nm with FWHM of 10 ± 2 nm) to reduce noise from background light.

### Flames

Measurements were performed at 6 mm height above the McKenna-type burner (HAB) in 15 different atmospheric CH_4_/N_2_/O_2_ laminar flame product zones with equivalence ratios from 0.6 to 1.5. The operating conditions for different flames can be found in Table II. The burner has a plug diameter of 64 mm and the water-cooled burner head was utilized to provide a flat premixed flame. The combustion products at HAB = 6 mm of different flames are simulated using ANSYS Chemkin-Pro 2020 R2 (with Gri-Mech 3.0)^43^ for the species composition information within the measured volume required by IR-LITGS thermometry.

**Table II.** Operating conditions for flames 1–15. ϕ is the equivalence ratio, and Q is the flow rate (L·min^–1^). T (K), M/γ (g·mol^–1^), α (mm^2^·s^–1^), and c_s_ (m·s^–1^) are the simulated flame temperature, mass-to-specific-heat ratio, thermal diffusivity, and speed of sound, respectively, in the products zone simulated using Chemkin.

Flame number	Operating conditions				Flames simulations			
	ϕ	*Q*_CH4_	*Q*_O2_	*Q*_N2_	*T*	*M*/γ	α	*c*_s_
1	0.6	0.79	2.64	7.00	2003.46	22.77	546.66	855.40
2	0.6	1.15	3.83	7.00	2283.05	23.10	686.42	906.45
3	0.7	1.30	3.71	9.00	2229.83	22.88	658.54	900.08
4	0.7	1.60	4.57	10.00	2297.21	22.94	695.30	912.52
5	0.8	1.82	4.55	10.00	2387.81	22.86	749.45	931.96
6	0.8	2.15	5.37	12.00	2375.40	22.84	742.43	929.86
7	0.9	2.25	5.00	12.00	2401.68	22.68	763.18	938.23
8	1.0	2.60	5.20	15.00	2319.98	22.45	722.55	926.84
9	1.1	2.40	4.36	15.00	2141.28	22.02	642.38	899.13
10	1.2	1.65	2.75	13.00	1855.38	21.39	518.19	849.18
11	1.3	1.30	2.00	9.00	1838.08	21.03	528.68	852.49
12	1.3	1.60	2.46	8.00	2032.55	21.30	625.06	890.68
13	1.4	1.50	2.15	7.00	1980.05	20.89	619.63	887.76
14	1.5	1.40	1.86	7.00	1858.89	20.38	577.28	870.91
15	1.5	1.10	1.47	5.00	1884.64	20.54	586.61	873.47

## Results and Discussion

### Laser-Induced Thermal Grating Spectroscopy Temperature Measurement in Flames

**Figure 2.**
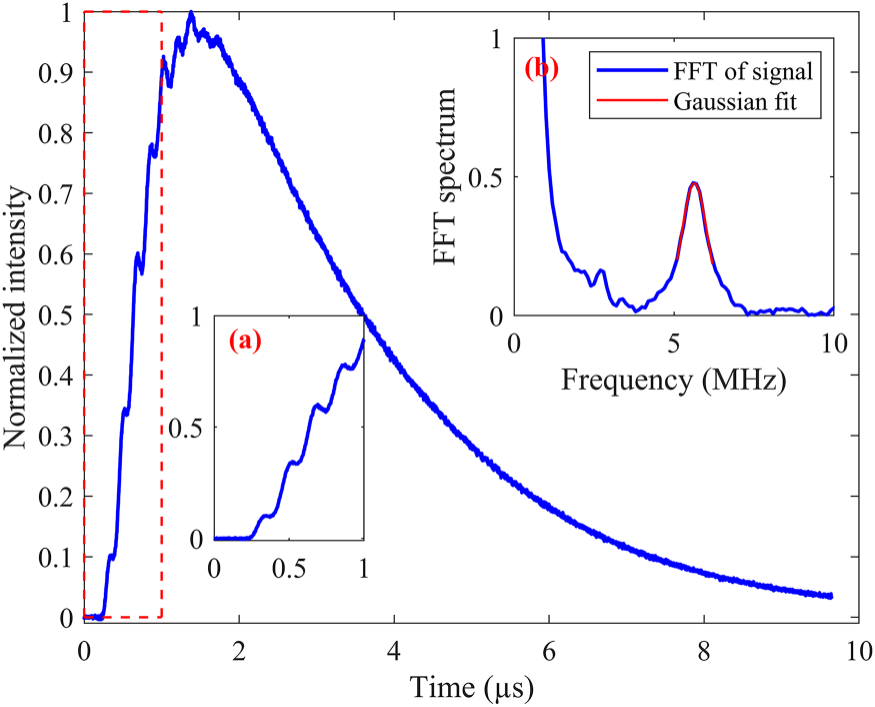
500 shot-averaged LITGS signal of 2000 ppm C_2_H_2_ diluted in N_2_ at room temperature with the pump lasers aimed at 3230.98 cm^–1^. Insert (a) zoom in the signal from 0 to 1 µs and insert (b) is the FFT of the signal. The Gaussian fit of FFT indicated a peak at *f*_osc_* *= 5.638 MHz.

The oscillation frequency of the LITGS signal can be determined either by fitting the experimental LITGS signal waveform to the simulation model, or by a Fourier transform. The extracted oscillation frequency is used to determine the speed of sound in the crossing volume as *c*_s _= *f*_osc_Λ. The grating spacing Λ, which depends on the exact crossing angle between the two pump beams, can be accurately obtained by a calibration measurement in an environment with a known speed of sound. Figure 2 shows the LITGS signal of 2000 parts per million (ppm) C_2_H_2_ diluted in N_2_ at 298 K. The oscillation frequency *f*_osc_ was determined to be 5.638 MHz in the calibration measurement. By using the speed of sound of pure N_2_ *c*_s_ = 353.9 m·s^–1^ at 298 K and 1 atm,^44^ the LITG spacing of this setup is determined as Λ = *c*_s_/*f*_osc_ = 62.77 µm. Note that the geometry of the crossing pump laser beams would normally give a grating spacing of about 125 μm but aligning the probe laser at the second Bragg angle causes diffraction of an overtone of the LITG, which behaves as a LITG with half the grating spacing of the original LITG.

**Figure 3.**
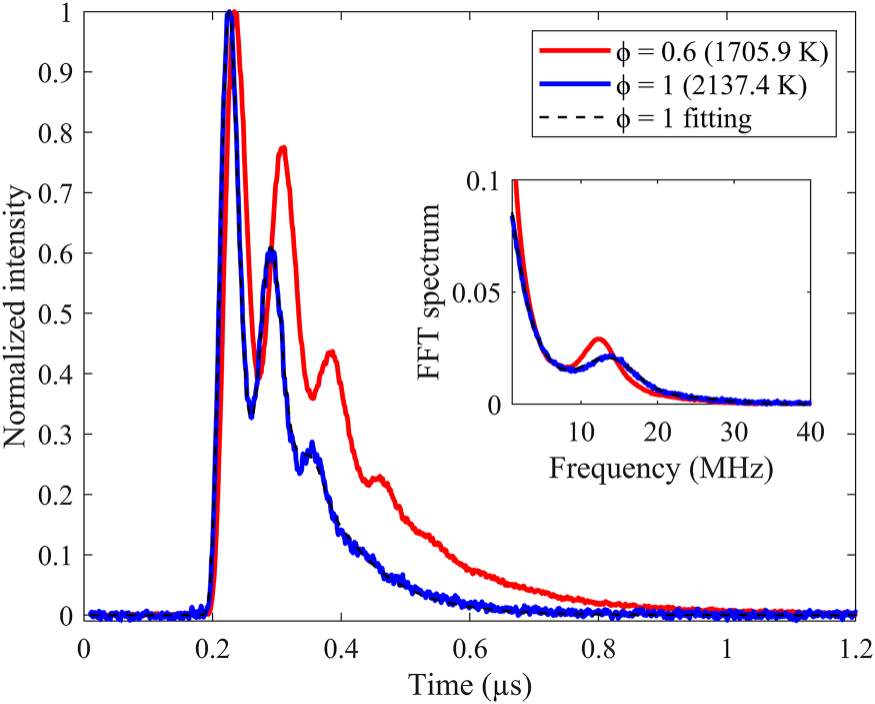
100 shot-averaged LITGS signal of water at flame temperature with the pump lasers aimed at 3230.98 cm^–1^ and theoretical temporal profile fitting of LITGS signal. Insert is the FFT of the signal.

Since quite a high S/N LITGS signal in flames can be achieved using 100 shot-averaging and longer time averaging has little effect on the oscillation frequency extraction from the temporal waveform, 100 shot-averaged LITGS signals were recorded in all flames for temperature measurement and the example signals at flame no. 1 (ϕ = 0.6) and flame no. 8 (ϕ = 1) are shown in Figure 3. Compared to the signal waveform in diluted C_2_H_2_ at room temperature, the flame conditions signal decay much faster due to the increased sound speed and thermal diffusivity, which is why the theoretical model is used to determine the oscillation frequency in the flame signals with better precision than the Fourier transform method.^29^ Generally, longer-lasting signals give higher precision, which is why the LITGS thermometry precision increases at high pressures.^32^ The contrast of the oscillations in the LITGS signals is caused by the rates of vibrational energy transfer of these two species:^39^ A fast initial energy transfer gives strong oscillations as seen in the H_2_O signals in Figure 3, while a slower energy transfer gives small oscillations on top of a stationary background as in the calibration signals in Figure 2.

**Figure 4.**
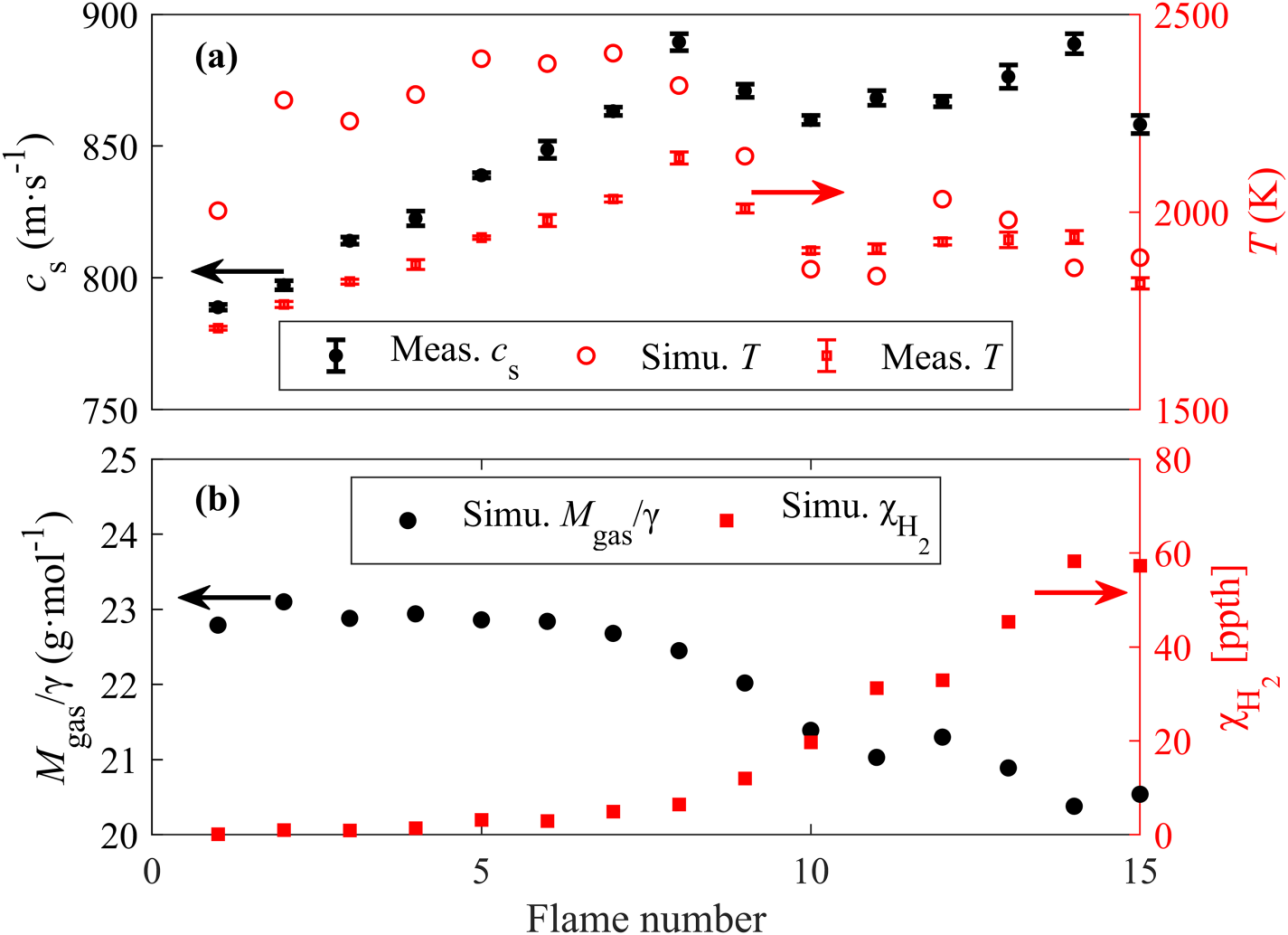
(a) Speed of sound and temperature measured with LITGS at 6 mm HAB for different flames, together with the simulated temperature. (b) Simulated *M*_gas_/γ and H_2_ concentration with Chemkin.

As shown in Figure 3, the LITGS signal at stoichiometric flame has a higher oscillation frequency due to the faster speed of sound at higher temperatures. With the knowledge of pre-calibrated LITG spacing Λ, the speed of sound *c*_s_ can be accurately determined from the LITGS oscillation frequency *f*_osc_ in various flames, as shown in Figure 4a. The precision of the *c*_s_ measurement, indicated by the error bars in Figure 4a, was estimated using the standard deviation of 10 repeated LITGS measurements, each recorded as an average of 100 single-shot signals. For these measurements, the precision is better than 0.5% (≤ 4.5 m·s^–1^).

For ideal gases, the relation between temperature *T* and sound speed can be expressed as *T* = *c*_s_^2^*M*_gas_/γ*R*_u_, where *R*_u_ is the universal gas constant and *M*_gas_/γ = (∑*_i_* χ*_i_M_i_*)/(∑*_i_* χ*_i_* γ*_i_*) is the mass-to-specific-heat ratio for gas mixtures. Thus, the gas temperature can be derived from *c*_s_ if the gas constants in the measurement volume can be estimated. In this work, the mole fractions of major combustion products χ*_i_* in different flames were taken from Chemkin simulation results based on Gri-Mech 3.0,^43^ together with heat capacity ratio for each species γ*_i_* as a function of temperature and molecular weight for each species *M_i_*, as shown in Figure 4b. 

The uncertainty in the gas composition, and hence the calculated *M*_gas_/γ in the measured volume, will affect the temperature measurement accuracy. For fuel-lean methane flames with equivalence ratios from 0.6 to 1.0 (flame no. 1 to flame no. 8), the mean value of *M*_gas_/γ of the major combustion products mixture shows little variation (changing from 22.77 g·mol^–1^ to 22.45 g·mol^–1^ from flame no. 1 to flame no. 8 according to simulation) since the major products have similar molecular weight and heat capacity ratio. Based on this, we assume a 1.5% uncertainty in the gas constants in the lean flames. This can be even further improved if *M*_gas_/γ in the measured volume can be more accurately determined. The flame temperature measurement precision in the product zone of the lean flames was estimated to be better than 0.8% (≤16 K), which was calculated by considering the standard deviation of 100 repeated temperature measurements. This high precision and accuracy demonstrate the suitability of IR-LITGS thermometry in lean flames. However, for fuel-rich methane flames with equivalence ratios from 1.1 to 1.5 (flame no. 9 to flame no. 15), the value of *M*_gas_/γ in the product zone decreases dramatically with higher ϕ, mainly due to the increasing H_2_ concentration. As a combustion product in fuel-rich flames, H_2_ has a very small molecular weight and makes a dominating contribution to the change in *M*_gas_/γ, as shown in Figure 4b. This means an accurate determination of the H_2_ concentration is crucial for accurate LITGS temperature measurements in rich flames.

### Acoustic Transit Time τ_tr_ and Thermal Diffusion Decay Time τ_th_

**Figure 5.**
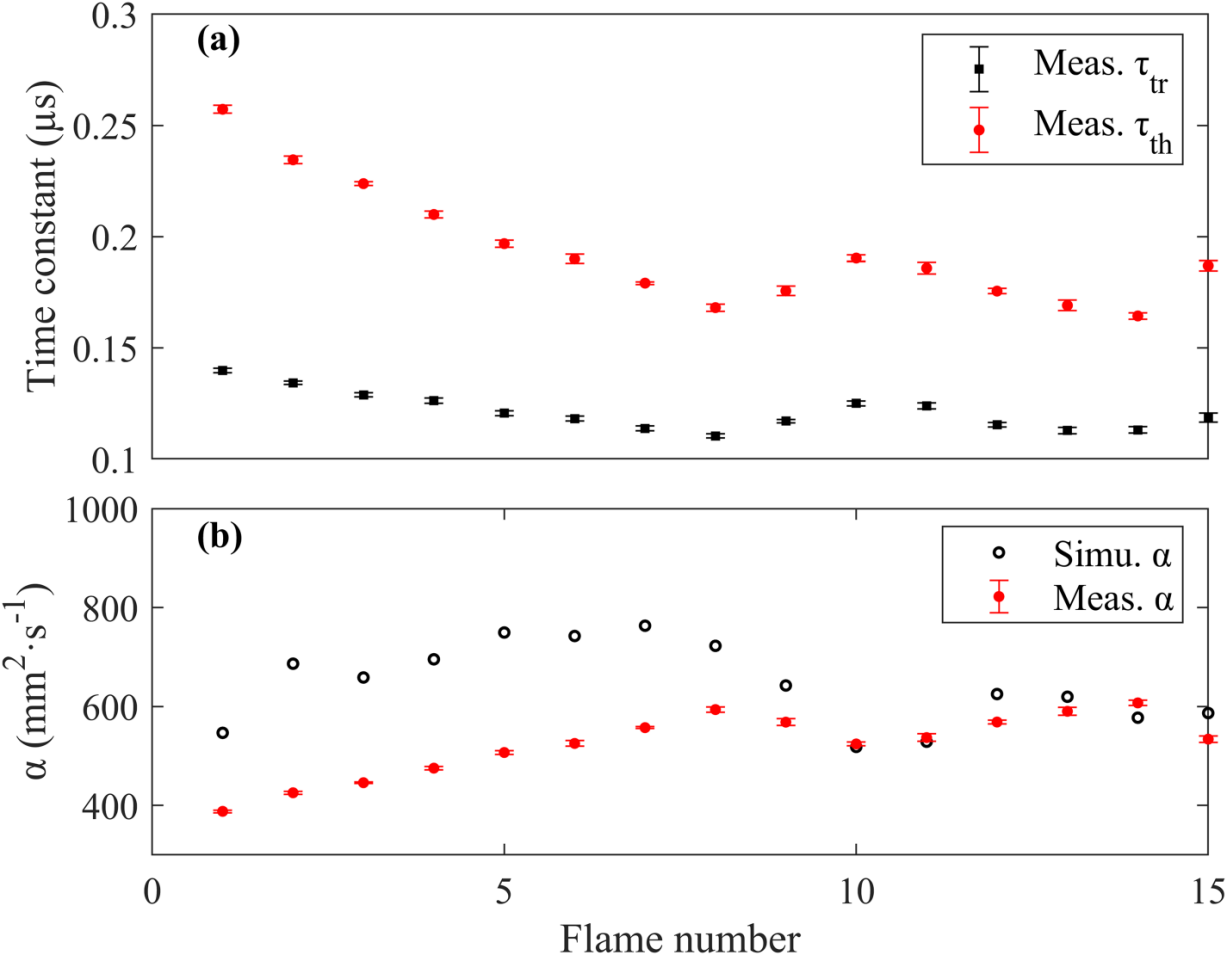
(a) The acoustic transit time τ_tr_ and thermal diffusion decay time τ_th_ measured in different flames. (b) Measured and simulated thermal diffusivity.

In addition to the oscillation frequency, time constants such as acoustic transit time and thermal diffusion decay time can also be extracted from LITGS waveform with very high precision better than 2% (≤3 ns) thanks to the high S/N (∼1000) of the signal and the signal waveform's insensitivity to the pulse laser intensity fluctuation, as shown in Figure 5a. 

The acoustic transit time τ_tr_ = *w*/( $\sqrt{2}$ *c*_s_) will be inversely proportional to the speed of sound since the beam radius did not change during measurement. However, the determined beam radius *w* = $\sqrt{2}$ *c*_s_τ_tr_ changes from 139 to 156 µm in these measurements. This may be caused by flame fluctuations affecting the crossing and beam diameter of the pump beams in the measurement volume but is more likely related to the uncertainty in the fitting model, which reduces the accuracy of determining the transit time due to uncertain influence from other fitting parameters.^45^

With the measured thermal diffusion decay time τ_th_, LITGS provides an accurate direct method for measuring the thermal diffusivity α = (Λ/2π)^2^/τ_th_ in flame environments, as shown in Figure 5b. Compared to the simulated value of α in different flames shown in Table II, the results measured by LITGS show an underestimation of 20%. This is reasonable since the thermal diffusivity is positively correlated with temperature, and the simulation calculates α based on the adiabatic flame temperatures, which are higher than the actual flame temperatures in this case. The thermal diffusivity measured from the LITGS signal can be used for accurate, in situ pressure measurements.^45^ Since the thermal diffusivity α is also temperature dependent, as can be seen in Figure 5(b), this could potentially provide a secondary high-precision flame temperature measurement from the LITGS signal independent of the oscillation frequency. This could be useful in high-temperature, low-pressure situations where the fast decay of the signal decreases the number of visible oscillations, and thereby decreases the precision of the measured speed of sound from the oscillations.

### Temperature Measurements in Rich Flames Using Degenerate Four-Wave Mixing

**Figure 6.**
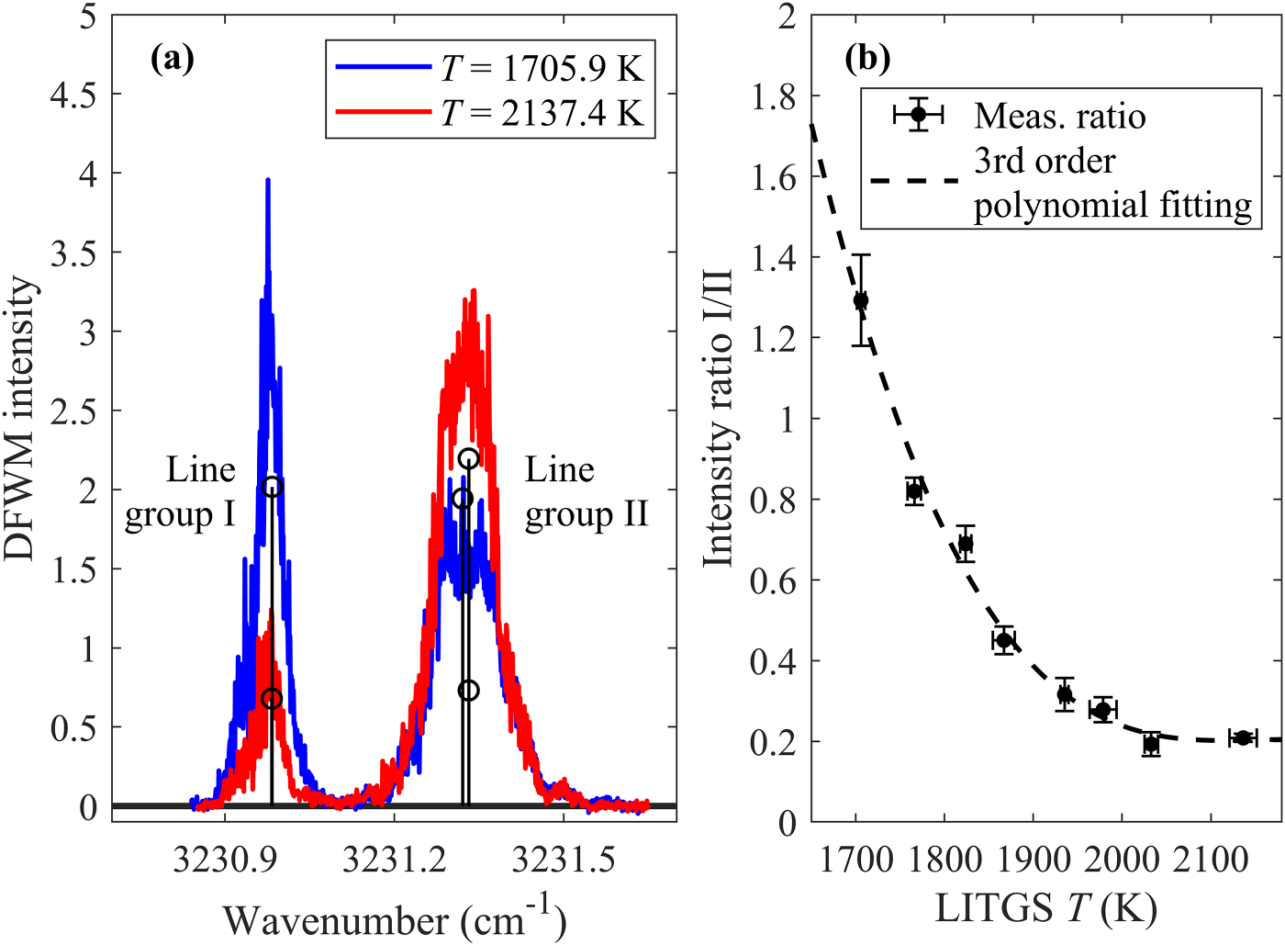
(a) DFWM spectrum of H_2_O at flame no. 1 and flame no. 8, scanned near 3231 cm^–1^. The stems indicate the individual transitions in line groups I and II specified in Table I, and the height represents relative line strength at 1800 K taken from HITEMP 2010.^42^ (b) The DFWM intensity ratio of line group I/II as a function of the flame temperature measured by LITGS. The error bars of temperature indicate the standard deviation of 100 repeat LITGS measurements, and the error bars of the area ratio are the standard deviation of 5 repeated DFWM scans over line group I/II.

Figure 6a shows the recorded IR-DFWM spectrum at 6 mm height above the burner in flame nos. 1 and 8, which demonstrates the feasibility of the DFWM water line thermometry by showing the dramatic change in the relative signal intensity between line groups I and II for different flame temperatures. Quantitative measurement of temperature using a DFWM signal intensity ratio requires a calibration of the degree of excitation saturation, and the measured temperatures in the lean flames (flame no. 1 to flame no. 8) using LITGS are taken as reference temperatures. The calibration of the relation between signal intensity ratio and temperature is achieved by recording the DFWM signal ratios at the same point in the laminar lean flames, as shown in Figure 6b, which will be further used for the quantitative DFWM temperature measurement in rich flames.

**Figure 7.**
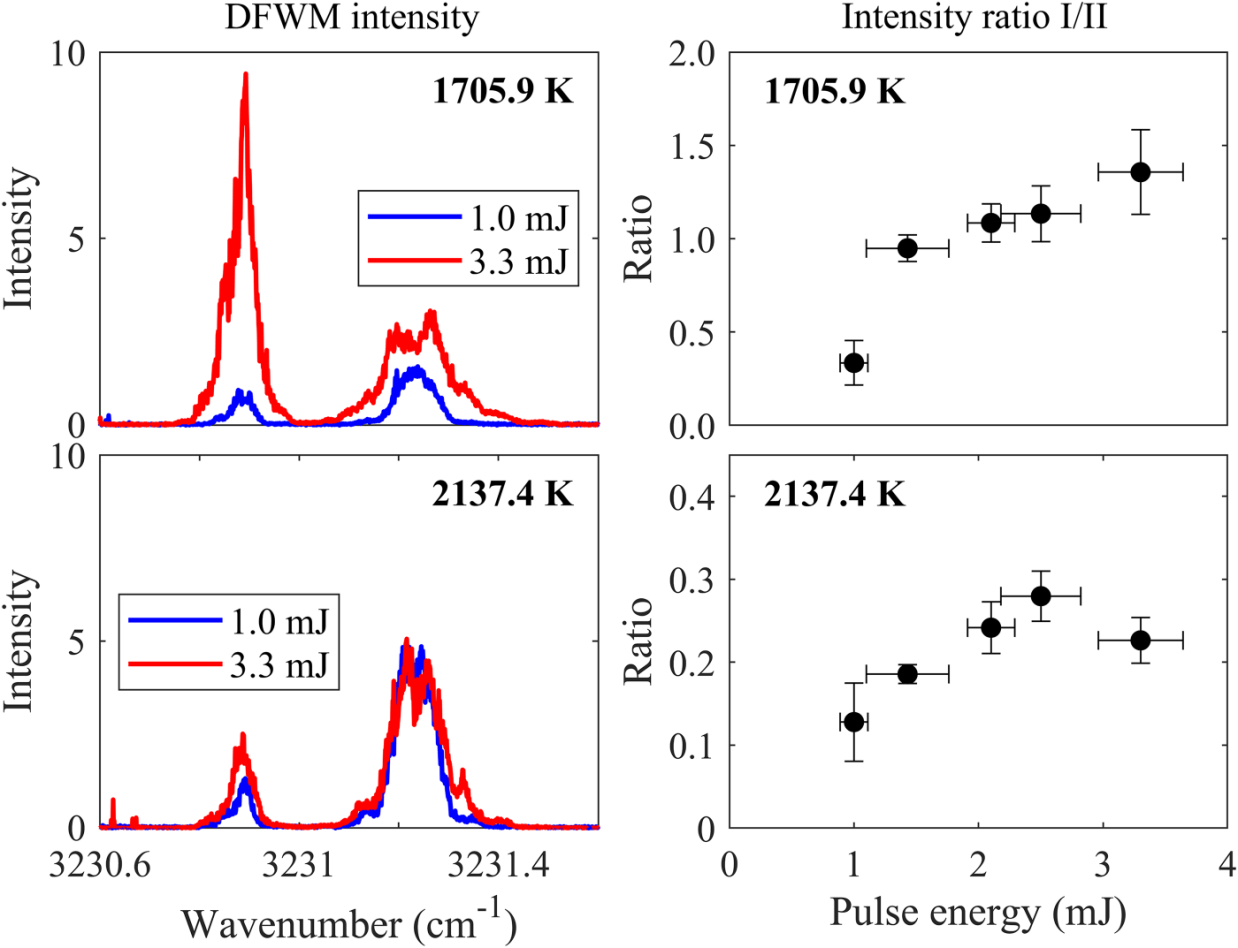
Saturation measurement in DFWM shows the signal ratio dependency on the total laser pulse energy at flame no. 1 and flame no. 8. The laser pulse energy used in the DFWM thermometry in this work is 3.3 mJ.

The degree of saturation during DFWM measurement in flames is also studied under this setup. As shown in Figure 7, within the temperature range involved in this work, when the laser pulse energy ≥ 2 mJ (in this work, the laser pulse energy is 3.3 mJ) the line ratio approaches a constant value with increasing laser energy, indicating the saturation condition is satisfied and the temperatures at lean flames can be used as calibration for the line ratio also in the rich flames.

### Flame Temperature Measurements in Lean and Rich Flames Using Combined LITGS/DFWM

**Figure 8.**
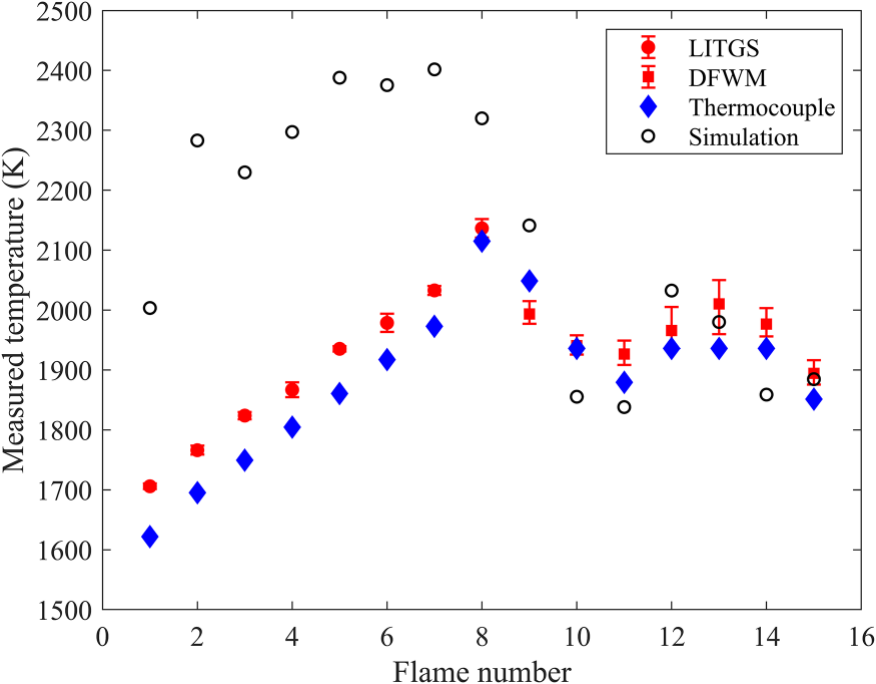
Simulated and measured flame temperatures by LITGS (flame nos. 1–8), DFWM (flame nos. 9–15), and thermocouple.

The flame temperatures measured with LITGS (flame no. 1 to flame no. 8) and DFWM (flame no. 9 to flame no. 15) are shown in Figure 8. As a comparison, the flame temperature was also measured with a thermocouple. It can be seen that the measured temperature in different flames is in good agreement with the trend shown in the thermocouple measurements, especially for the LITGS data in lean flame. The thermocouple seems to underestimate the flame temperatures compared to the LITGS and DFWM temperatures. As the thermocouple used does not have high accuracy, and the LITGS temperatures have proved to be very accurate in lean flames,^29^ this is assumed to be due to a systematic error in the thermocouple measurement.

The DFWM temperature measurements show less precision (≤90 K) compared with LITGS temperature data. This is partly due to laser intensity fluctuations during the DFWM excitation scan during scanning (one scan typically takes several minutes), and partly due to the shot-to-shot variations in the mode structure of the infrared laser beam, which originates in the dye laser. These fluctuations during the scan result in fluctuations in the DFWM line intensity ratio in different scans which is why the DFWM temperature is calculated as the average of five consecutive scans. The LITGS method, which measures temperature based on temporal profile and frequency, is less sensitive to the fluctuation of laser energy and mode structure, which leads to higher precision in the LITGS temperature measurement.

## Conclusion

In this work, the combined IR-LITGS and IR-DFWM techniques are demonstrated for precise, spatially resolved thermometry in premixed CH_4_/O_2_/N_2_ flames (equivalence ratios from 0.6 to 1.5), by probing the rovibrational transitions of hot water near 3231 cm^–1^. As a comparison, the flame temperature was also measured with a thermocouple. The measured temperatures in different flames are in good agreement with the trend shown in the thermocouple measurements, especially for the LITGS data in lean flames. In lean flames, the temperature was measured using LITGS signals with pump lasers targeting the hot water line group at 3230.98 cm^–1^, with a precision better than 16 K (0.8%). In rich flames, the temperature was measured by recorded DFWM excitation scans from 3230.85 to 3231.6 cm^–1^, in which the signal intensity ratio between the covered two hot water line groups shows a sensitive temperature dependency. However, since the ratio is also affected by experimental arrangements and laser energy levels, accurate temperature measurement requires calibration of the relationship between temperature and ratio in advance. The high-precision flame temperatures measured with LITGS at the same position were used to calibrate the DFWM line intensity ratio versus the temperature in the lean flames, and this calibration curve was later used for DFWM thermometry in the rich flames. In addition, using LITGS temperature as calibration is also an improvement since the similarity between the LITGS and DFWM setup means the temperature measurements can be performed simultaneously using the same pump laser system. The precision in the DFWM temperature measurement was estimated to be better than 90 K in this work. Although the laser energy in this DFWM measurement is enough to saturate the absorption, it is found that the spectrum of DFWM is still affected by the shot-to-shot-fluctuations in intensity and mode structure of the pump laser during measurement, which will reduce the DFWM temperature measurement accuracy. Compared to DFWM thermometry, the frequency-based LITGS thermometry method has better potential for high-precision temperature measurements since it is unaffected by pulse-to-pulse fluctuations in the pump laser. In addition to temperature, the temporal shape of the LITGS signal can also be used to determine other physical quantities, such as the local speed of sound and thermal diffusivity, by precisely fitting a simulated LITGS signal to the measured time waveform. The precision in the fitting is better than 0.5% and 1.3% for the speed of sound and thermal diffusivity, respectively. These measurements can provide more data for flame simulations. Also, the thermal diffusivity can be used for in situ pressure measurements or, if the pressure is constant, the temperature dependence of the thermal diffusivity can be used as another flame temperature from the LITGS signal.

This combined LITGS/DFWM thermometry is applicable in laminar flames or in quasi-stable combustion where the flame conditions stay stable over several minutes. Applications in turbulent flame situations are not possible at this time, because the time resolution is limited by the scanning speed of the DFWM laser system, where scanning over the lines takes a few minutes. It is possible to improve this time resolution by recording the peak intensity of each line instead of scanning the laser,^6,31^ but it is still necessary to average the intensity of each line over 30 s to reduce the shot-to-shot intensity fluctuations,^6^ and for laminar flames the scanning over each line gives a higher precision.^20^ It might be possible to achieve better time-resolution in the future by having a laser system where the laser wavelength can be switched between the H_2_O lines from shot-to-shot with higher repetition rate, but at this point the time resolution is limited to ∼1 min per measurement and is therefore applicable only in laminar, quasi-stable flame conditions.

In summary, the LITGS thermometry method has higher precision and the capacity for single-shot flame thermometry and has high accuracy in situations where the gas composition can be estimated. For thermometry in less-known flame situations, for example, during biomass combustion,^6^ the DFWM water line ratio method can be applied to thermometry and combined with LITGS it is possible to achieve an accurate calibration of the line intensity ratio for the current level of saturation. Since mid-infrared DFWM can also be used for concentration measurements of many important pollutants, fuels, and intermediate species, this opens up the field for simultaneous concentration and temperature measurements for investigations of new biofuels and other thermochemical reactions in quasi-stable laminar flame conditions.
